# Reliability of isokinetic knee strength measurements in children: A systematic review and meta-analysis

**DOI:** 10.1371/journal.pone.0226274

**Published:** 2019-12-17

**Authors:** Laura Muñoz-Bermejo, Jorge Pérez-Gómez, Fernando Manzano, Daniel Collado-Mateo, Santos Villafaina, José C. Adsuar

**Affiliations:** 1 University Centre of Merida, University of Extremadura, Mérida, Spain; 2 Health, Economy, Motricity and Education Research Group, Faculty of Sport Sciences, University of Extremadura, Cáceres, Spain; 3 Faculty of Sport Science, University of Extremadura, Cáceres, Spain; University of Mississippi Medical Center, UNITED STATES

## Abstract

Measuring muscle strength using isokinetic dynamometry allows evaluating and comparing normal and sick children, establishing recovery and rehabilitation goals, and quantitatively monitoring the course of a disease and the response to treatment. The purpose of this study was to carry out a systematic review and meta-analysis focusing on studies that examined the test-retest reliability of isokinetic knee strength measurements in children. This study is important because isokinetic dynamometry is the gold standard for evaluating muscle strength and it allows comparing muscle performance in children. The databases used were PubMed, Web of Science Scopus, and Embase (up to July 26, 2019). Only studies published in English were included in this review. All studies focused on the reliability of isokinetic knees in healthy children or those with cerebral palsy applied to dynamic contractions (concentric or eccentric) and provided measures of strength, reproducibility, ICC, peak torque, or SEM. We found a total of 143 abstracts and examined 94 articles to determine if they met the inclusion criteria. Finally, 10 articles were included in the systematic review and five studies (96 subjects) formed the meta-analysis sample, all of which focused on the reliability of isokinetic knees in the concentric mode. The CAT and QAREL scales were used to assess the quality of the included studies. The meta-analysis revealed high intra-class correlation coefficients (ICC) (0.84; *p* < 0.001; n = 96 subjects) in the flexion and excellent intra-class correlation coefficients (ICC 0.90; *p* < 0.001; n = 96 subjects) in the extension. Isokinetic dynamometry could be indicated as a method for measuring muscle strength training in children. However, the reviewed studies suggest some methodological issues in isokinetic tests, such as the rest days between testing and retesting, using the same speeds, protocols, and evaluators, and the performance of the subjects, so more research is required.

## Introduction

The term “muscle strength” refers to a muscle's or a group of muscles’ ability to exert maximum muscular force [1]. Isokinetic dynamometry is the gold standard in muscle strength evaluation [2]. It is an adequate system for assessment and diagnosis in the field of biomechanics. Measuring muscle strength allows evaluating and comparing normal and diseased children, establishing goals for recovery and rehabilitation, and quantitatively monitoring the course of a disease and the response to treatment [3]. Therefore, following the safety standards and recommendations for children, isokinetic dynamometry is safe [4] and allows registering a range of angular velocities in both concentric and eccentric exercises [5].

Reliability is defined as the extent to which measurements can be replicated [6]. Reliability can be presented in relative or absolute values. Relative reliability indicates the degree to which individuals maintain their position in a sample with repeated measurements [7]; the most common indicator of relative reliability is intra-class correlation coefficients (ICC) [8]. In contrast, absolute reliability refers to the degree of conformity of the measures of a test from moment to moment [4]. The most common indicators of absolute reliability are the standard error of measurements (SEM) and the smallest real difference (SRD).

The reliability of measuring isokinetic knee strength with a dynamometer in children has not been fully investigated. The majority of isokinetic strength reliability studies report the correlation coefficient as an indicator of the agreement between measurements [1]. When reported, often only relative reliability is addressed, via ICC [9]. The relative reliability of isokinetic knee strength measurements in this age group has been reported as moderate [2, 4] to high [10, 11], and this parameter has been given more importance in previous studies. However, absolute reliability, which refers to the degree of conformity in test measurements from one time-point to another, has not yet been determined in depth. This should be the next step for these studies to clarify clinically important changes for patients [4]. Only two previous studies [4, 12] have given an approximation for the value of SRD. The present review presents more information on this variable. For the above reasons, it is important to know the reliability of isokinetic dynamometry.

Scattered information is available on the reliability of isokinetic measurements for children. Moreover, to our knowledge, no systematic reviews or meta-analyses on this topic have been published. Therefore, the purpose of this research was to perform a systematic review and meta-analysis of existing values of the test-retest reliability of isokinetic knee strength measurements in children, discuss potential limitations of the literature, and suggest recommendations for future research on statistical analyses for interpreting reliability. The current review and meta-analysis can provide valuable information for future guidelines and strategies for muscle strength reeducation in children.

## Materials and methods

This systematic review meets the Preferred Reporting Items for Systematic Reviews and Meta-Analyses (PRISMA) guidelines (S1 File).

### Eligibility criteria

Test-retest studies published in English (up to July 26, 2019) that measured knee flexion and extension in children using an isokinetic dynamometer were included.

For this research, we applied further eligibility criteria. Eligible studies were those that assessed the reliability of isokinetic knees in healthy children or those with cerebral palsy applied to dynamic contractions (concentric or eccentric) and that provided measures of force, reproducibility, ICC, peak torque, or SEM.

We did not include studies that reported duplicate results.

The meta-analysis included studies on the reliability of knee movement in the concentric mode, both for the extension and for the flexion of the joint at a speed of 60°/s, that always included the reliability values of the dominant leg of the subjects.

Two of the authors (FM and JCA) independently examined and screened the titles and abstracts of the retrieved articles to assess study eligibility. Any disagreement or uncertainty was resolved through discussion. All reviewers reviewed the full-text articles that met the inclusion criteria or had uncertain eligibility. Any disagreement was resolved by consensus.

### Electronic literature search

Several databases were searched, including PubMed, Web of Science, Scopus, and Embase.

A combination of MeSH terms was used for the PubMed search: child, knee, muscle strength, reproducibility of results, and validation studies.

The specific keywords used for the Web of Science, Scopus, and Embase were combinations of “isokinetic”, “knee”, “reliability”, “reproducibility”, “children”, “kids”, “boys”, “girls”, and “dynamometer”. The exact search was: *((children [Title/Abstract] OR kids [Title/Abstract] OR boys [Title/Abstract] OR girls [title/abstract]) AND knee [Title/Abstract] AND (reliability [Title/Abstract] OR retest [Title/Abstract] OR test-retest [Title/Abstract] OR reproducibility [Title/Abstract]) AND isokinetic [Title/Abstract])*.

After examining the records, additional searches were conducted in health improvement sources, meta-search engines (Google/Google scholar), and on the Retraction Watch website to identify additional publications and gray literature.

### Isokinetic dynamometry

Isokinetic movement is defined by maintaining an angular velocity of constant movement throughout the joint path. The current isokinetic dynamometry system allows evaluating in both concentric and eccentric modes of exercise. The dynamometer shows the value of the moment of force developed at each instant. The most important data recorded by the isokinetic dynamometer is the peak torque or maximum moment of force, which indicates the highest value of force recorded during the test. Another variable an isokinetic dynamometer provides is “work”, which expresses the product of the moment of force and angular distance. Peak torque was used in this review.

### Evaluation of the quality of the included studies

The selected studies were evaluated using the clinical evaluation tool (CAT) scale developed by Brink and Louw [13] and the Quality Appraisal for Reliability Studies (QAREL) [14].

The CAT scale is an instrument developed specifically to evaluate the methodological quality of studies, considering the validity and reliability of the objective clinical tests. The CAT scale contains 13 evaluation items. Four of the 13 items refer to validity issues, but the other nine refer to reliability; therefore, only these nine were used for this review. Each article was classified as "yes" when information was described in sufficient detail or "no" when there was not enough information for clarification [13]. A final percentage (%) evaluation column was added based on the items that each study achieved. Thus, the maximum possible score was 90%, which represents the highest methodological quality. Studies were considered high quality if they scored above 45%.

The Quality Appraisal for Reliability Studies (QAREL) scale is a quality assessment tool for diagnostic reliability studies. It consists of 11 questions (meets, does not meet, doubtful or not applicable) grouped into three categories of internal validity (items 3–9), external validity (items 1, 2, and 10), and the relevance of the statistical analyses (item 11). The maximum score is 110%.

### Statistical analysis

The meta-analysis included only five articles [2, 4, 10–12] of the ten incorporated in this review. This meta-analysis focused on the reliability of the concentric mode, both for extension and knee flexion. The choice of these items was because the studies all used a similar speed, 60°/s, always with the values of the dominant leg of the subjects. In addition, we considered the sample size and the type of participants; all were healthy except for those in Moreau’s study, whose subjects had cerebral palsy (CP) [10].

Heterogeneity between the included studies was assessed using the chi-squared test on Cochran’s Q (alpha set at 0.1) statistic [15] and Higgins and Thompson's I^2^ statistic [16].

The random-effects model and the fixed-effects model were used to combine standardized effect sizes with a 95% confidence interval.

The funnel plot and Egger´s weighted regression tests were used to evaluate possible publication bias (p ˂ 0.1 was considered statistically significant publication bias).

Regarding the reliability indicators used in this review, we started with the ICC, which is usually the main indicator of reliability. All ICCs collected in our review are shown at a 95% confidence interval (CI) [12].

The SEM was calculated from the square root of the mean error term derived from the analysis of variance (ANOVA) [12] and was used to determine the minimum difference that is considered important for a single subject [4]. SEM was calculated as $SEM=SD\sqrt{\left(1-ICC\right)}$, where SD is the standard deviation of day 1 and day 2 [17].

The SRD is shown as a measure of sensitivity to change. In the original formulation, SRD was defined as the 95% confidence limit of the SEM of difference scores [18]. The SRD was calculated as $SRD=1.96\times \sqrt{2}\times SEM$ [17].

The percentages of SEM and SRD are shown to represent the error of measurement in relative terms and thus allow comparing the different variables [12]. This was calculated with: [SEM or SRD / mean of all values] [12].

## Results

### Search strategy and quality of studies

**Fig 1:**
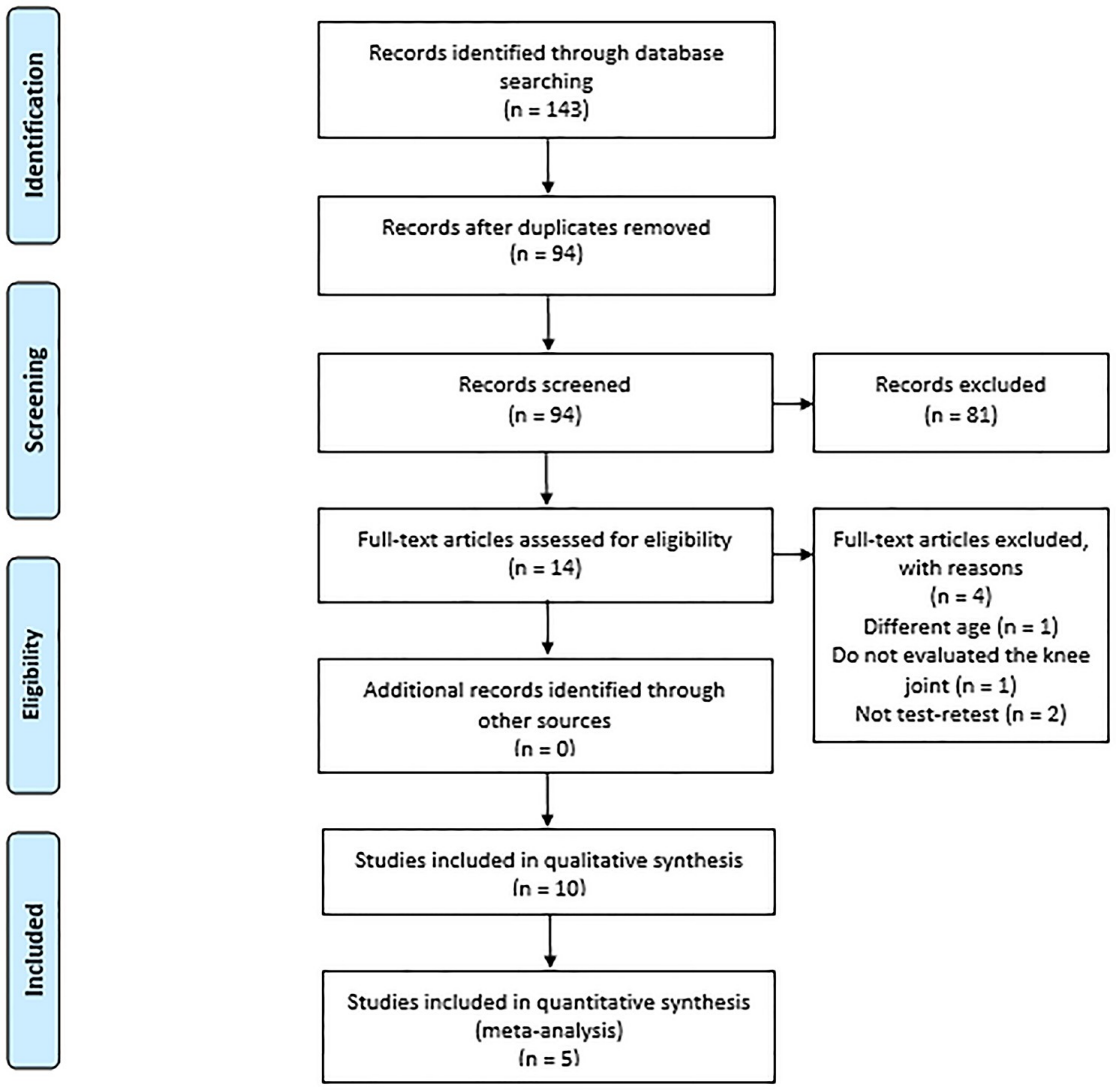
Flow diagram of the search process and selection of documents for the review.

A total of 143 studies were identified through searches in electronic databases. Among these studies, we identified and eliminated 49 duplicates. Only 14 studies met the inclusion criteria, but four of these studies were excluded. One study was excluded because it presented different ages, another because it did not evaluate the knee joint, and another two because they did not perform test-retest. A manual search of the bibliographies of the relevant articles revealed no additional studies. Therefore, ten prospective studies were included in this systematic review on isokinetic knee movement in children (Fig 1).

The quality of the articles according to the CAT quality score varied between 23% and 78%, with the maximum possible being 90%. According to the analysis, eight articles were evaluated as high quality (Table 1). The quality of the articles varied according to the designs of the studies.

**Table 1:** Evaluation of the quality of the studies with clinical evaluation tool (CAT).

Study	1	2	3	4	5	6	7	8	9	%
Ayalon et al. (2000)[27]	yes	yes	no	yes	no	yes	yes	no	yes	67
Fagher et al. (2016)[4]	yes	no	no	yes	no	yes	yes	no	yes	56
Iga et al. (2006)[5]	yes	no	no	no	no	yes	yes	no	no	34
Johnsen et al. (2015)[12]	yes	yes	yes	yes	no	yes	yes	no	yes	78
Kellis et al. (1999)[11]	yes	no	no	yes	no	yes	yes	no	yes	56
Merlini et al. (1995)[3]	yes	no	no	yes	no	yes	yes	no	yes	56
Moreau et al. (2008)[10]	yes	no	no	yes	no	yes	yes	no	yes	56
Pierce et al. (2006)[20]	yes	no	no	yes	no	no	yes	no	yes	45
Santos et al. (2013) [2]	yes	yes	no	yes	no	yes	yes	no	yes	67
Van den Berg-Emons et al. (1996)[21]	yes	no	no	no	no	no	yes	no	no	23

%: (Items "yes" x 100)/9; 1. If human subjects were used, did the authors give a detailed description of the sample of subjects used to perform the test? 2. Did the authors clarify the qualification, or competence of the rater(s) who performed the test? 3. If interrater reliability was tested, were raters blinded to the findings of other raters? 4. If intrarater reliability was tested, were raters blinded to their own prior findings of the test under evaluation? 5. Was the order of examination varied? 6. Was the stability (or theoretical stability) of the variable being measured taken into account when determining the suitability of the time interval between repeated measures? 7. Was the execution of the test described in sufficient detail to permit replication of the test? 8. Were withdrawals from the study explained? 9. Were the statistical methods appropriate for the purpose of the study? %: final percentage of reliability.

According to the QAREL scale, the quality of the articles varied between 30% and 100%, with the maximum possible being 110% (Table 2). Evaluation data indicate that eight articles scored between 60 and 100%.

**Table 2:** Evaluation of the quality of the studies with Quality Appraisal of Reliability Studies (QAREL).

Study	1	2	3	4	5	6	7	8	9	10	11	%
Ayalon et al. (2000)[27]	Yes	Yes	No	Yes	UC	Yes	No	No	Yes	Yes	Yes	70
Fagher et al. (2016)[4]	Yes	No	No	Yes	UC	Yes	No	No	Yes	Yes	Yes	60
Iga et al. (2006)[5]	Yes	No	No	No	No	Yes	Yes	No	Yes	Yes	No	50
Johnsen et al. (2015)[12]	Yes	Yes	Yes	Yes	Yes	Yes	Yes	No	Yes	Yes	Yes	100
Kellis et al. (1999)[11]	Yes	No	No	Yes	UC	Yes	No	No	Yes	Yes	Yes	60
Merlini et al. (1995)[3]	Yes	No	No	Yes	UC	Yes	No	No	Yes	Yes	Yes	60
Moreau et al. (2008)[10]	Yes	No	No	Yes	UC	No	No	No	Yes	Yes	Yes	60
Pierce et al. (2006)[20]	Yes	No	No	Yes	Yes	No	Yes	No	No	Yes	Yes	60
Santos et al. (2013) [2]	Yes	Yes	No	Yes	UC	Yes	No	No	Yes	Yes	Yes	70
Van den Berg-Emons et al. (1996)[21]	Yes	No	No	No	Yes	No	No	No	No	Yes	No	30

%: (Items "yes" x 100)/11; Was the test evaluated in a sample of subjects who were representative of those to whom the authors intended the results to be applied? 2. Was the test performed by raters who were representative of those to whom the authors intended the results to be applied? 3. Were raters blinded to the findings of other raters during the study? 4. Were raters blinded to their own prior findings of the test under evaluation? 5. Were raters blinded to the results of the reference standard for the target disorder (or variable) being evaluated? 6. Were raters blinded to clinical information that was not intended to be provided as part of the testing procedure or study design? 7. Were raters blinded to additional cues that were not part of the test? 8. Was the order of examination varied? 9. Was the time interval between repeated measurements compatible with the stability (or theoretical stability) of the variable being measured? 10. Was the test applied correctly and interpreted appropriately? 11. Were appropriate statistical measures of agreement used?

Yes; No; UC: unclear.

### Characteristics of the studies

Table 3 shows the characteristics of the participants. The sample sizes (n) of the final ten studies ranged from 12 to 39 participants, with participants aged 5–15  years. Some of the studies compared the dominant side with the non-dominant side. The joint evaluated in all studies was the knee. All studies evaluated the segments using computerized isokinetic dynamometers: Lido—Active, Cybex II, Cybex Norm, Biodex 6000, Biodex System 3, and Biodex System 4.

**Table 3:** Characteristics of the participant.

Study	N	Age	Gender	Type Subjects	Bilateral	Time of rest	Dynamometer
Ayalon et al. (2000)[27]	12	9–15	N/S	Cerebral Palsy	No	7 days	Cybex II
Fagher et al. (2016)[4]	22	8–10	N/S	Healthy	No	7 days	Biodex System 4
Iga et al. (2006)[5]	23	13–14	Boys	Healthy	Yes	7 days	Lido Active
Johnsen et al. (2015)[12]	28	12	N/S	Sports-active	Yes	7 days	Biodex 6000
Kellis et al. (1999)[11]	13	13	N/S	Footballers	Yes	7 days	Cybex Norm
Merlini et al. (1995)[3]	12	6–8	Boys	Healthy	No	3 days	Lido Active
Moreau et al. (2008)[10]	12	10–23	N/S	Cerebral Palsy	No	7 days	Biodex Medical System
Pierce et al. (2006) [20]	15	10–12	N/S	Cerebral Palsy	No	1 hour	N/S
Santos et al. (2013) [2]	21	5–12	N/S	Healthy	Yes	7 days	Biodex System 3
Van den Berg-Emons et al. (1996) [21]	12/39	6–12	N/S	Cerebral Palsy	No	1.5 hours	Cybex II

Age (years); N/S: Not Specified.

The contraction speeds ranged from 15°/s to 247.5°/s (4.32 rad/s) for the evaluation of the concentric mode and 60 to 180°/s for the eccentric mode.

To classify the strength of reliability, we used the scale proposed by Landis and Koch [19]: 0 represents poor reliability, 0.01–0.20 slight reliability, 0.21–0.40 regular reliability, 0.41–0.60 moderate reliability, 0.61–0.80 substantial reliability, and 0.81–1.00 very high (almost perfect) reliability.

All selected articles presented strength measurements for both knee flexion and extension in the concentric mode. Of these, only three [4, 5, 11] analyzed knee flexion and extension in the eccentric mode as well. Four studies [2, 5, 11, 12] included tests on both dominant and non-dominant legs. Studies found substantial and very high (almost perfect) reliability for all analyses, regardless of movement, speed, type of contraction, evaluation of the dominant or non-dominant leg, and whether the participants had CP or not.

The Pierce’s study identified regular and moderate reliability where the knee flexion was assessed at 15°/s and 90°/s with ICCs of 0.31 and 0.38, respectively. This study also identified moderate reliability, because it measured knee extension assessed at 15°/s and at 90°/s with ICCs of 0.51 and 0.50, respectively. Both movements were in the concentric mode [20]. Fagher also measured knee flexion at 60°/s and 180°/s with ICCs of 0.62 and 0.49, respectively. This study, although it showed substantial reliability when measuring knee extension at 180°/s with an ICC of 0.68 in the eccentric mode, showed moderate reliability (0.60) for knee flexion [4] (Table 4).

**Table 4:** Relative and absolute reliability of concentric extension and flexion of the knee in isokinetic.

Knee action evaluated	Speed (°/s)	Mean /(SD) 1° test	Mean /(SD) 2° test	Mean /(SD) 3° test	ICC (95% CI)	SEM (Nm)	SEM (%)	SRD (Nm)	SRD (%)
**CONCENTRIC**									
**EXTENSION**									
Fagher et al. (2016)[4]	60	47.8 (13.1)	51.0 (13.0)	-	0.81 (0.58–0.92)	5.5	11.1	15.3	30.9
	180	34.5 (9.6)	38.4 (7.9)	-	0.68 (0.30–0.86)	4.5	12.4	12.6	34.4
Johnsen et al. (2015)[12]	60	DL 104.0 (15.7)	DL 106.3 (15.4)	-	0.87 (0.73–0.94)	5.5	5.2	15.2	14.4
		NDL 101.9 (17.4)	NDL 106.7 (15.8)	-	0.85 (0.62–0.94)	5.6	5.4	15.5	14.9
Santos et al. (2013)[2]	60	DL 139.5 (36.8)	DL 139.8 (44.2)	-	0.87	15.8	11.2	-	-
		NDL 137.5 (34.8)	NDL 138.3 (49.3)	-	0.81	16.3	11.8	-	-
Moreau et al. (2008)[10]	60	39.24 (20.42)	37.42 (16.32)	-	0.95	-	-	-	-
Pierce et al. (2006)[20]	15	2.4 (1.6)	2.1 (1.7)	-	0.51	-	-	-	-
	90	7.1 (5.0)	5.2 (3.8)	-	0.50	-	-	-	-
	180	13.5 (9.8)	10.6 (9.8)	-	0.86	-	-	-	-
Merlini et al. (1995)[3]	100	40.1 (12.0)	41.2 (15.0)	40.4 (15.6)	0.95	-	-	-	-
		41.6 (13.4)	41.0 (15.3)	41.4 (14.1)	0.95	-	-	-	-
Kellis et al. (1999)[11]	60	DL 100.9 (12.3)	DL98.1 (12.1)	-	0.98	-	-	-	-
	120	DL 87.2 (12.1)	DL 85.1 (12.6)	-	0.96	-	-	-	-
	180	DL 74.7 (10.9)	DL 74.3 (11.9).	-	0.89	-	-	-	-
	60	NDL 98.8 (14.1)	NDL 9.5 (16.0)	-	0.96	-	-	-	-
	120	NDL 86.3 (13.0)	NDL 85.6 (14.0)	-	0.93	-	-	-	-
	180	NDL 71.8 (12.2)	NDL 73.1 (12.7)	-	0.94	-		-	-
Iga et al. (2006)[5]	1.08 rad/s	DL 159 (42)	DL 161 (44)	-	-	-	-	-	-
	2.16 rad/s	DL 133 (32)	DL 139 (35)	-	-	-	-	-	-
	4.32 rad/s	DL 106 (33)	DL 111 (34)	-	-	-	-	-	-
	1.08 rad/s	NDL 154 (44)	NDL 160 (47)	-	-	-	-	-	-
	2.16 rad/s	NDL 128 (29)	NDL 163 (34)	-	-	-	-	-	-
	4.32 rad/s	NDL 103 (26)	NDL 109 (29)	-	-	-	-	-	
Ayalon et al. (2000)[27]	90	30.52 (2.76)	30.39 (2.93)	-	0.98–0.99	-	-	-	-
Van den Berg-Emons et al. (1996)[21]	30	41.3 (15.7)	39.5 (16.7)	-	-	-	-	-	-
	60	39.4 (19.5)	39.8 (17.4)	-	-	-	-	-	-
	120	27.1 (9.7)	30.8 (12.8)	-	-	-	-	-	-
**FLEXION**									
Fagher et al. (2016)[4]	60	26.0 (5.5)	27.7 (6.2)	-	0.62 (0.28–0.82)	3.5	13.1	9.8	36.5
	180	20.4 (4.3)	23.9 (5.4)		0.49 (0.03–0.77)	3.1	13.9	8.5	38.5
Johnsen et al. (2015)[12]	60	DL 54.3 (11.0)	DL 55.1 (9.2)	-	0.81 (0.63–0.91)	4.4	8.1	12.2	22.3
		NDL 52.5 (10.5)	NDL 53.4 (9.0)	-	0.77 (0.55–0.89)	4.7	8.9	13.0	24.6
Santos et al. (2013)[2]	60	DL 106.9 (21.6)	DL 112.5 (29.1)	-	0.82	25.0	17.3	-	-
		NDL 113.9 (30.2)	NDL 118.0 (33.6)	-	0.79	15.0	12.8	-	-
Moreau et al. (2008)[10]	60	18.18 (14.57)	18.21 (13.63)	-	0.96	-	-	-	-
Pierce et al. (2006)[20]	15	0.2 (1.0)	0.7 (1,3)	-	0.31	-	-	-	-
	90	1.1 (2.0)	1.3 (1.4)	-	0.38	-	-	-	-
	180	6.1 (4.0)	4.8 (3.3)	-	0.80	-	-	-	-
Merlini et al. (1995)[3]	100	25.2 (8.7)	26.9 (8.3)	26.9 (7.5)	0.85	-	-	-	-
		27.3 (7.7)	26.7 (9.0)	26.6 (8.4)	0.85	-	-	-	-
Kellis et al. (1999)[11]	60	DL 64.5 (12.1)	DL 66.8 (8.8)	-	DL 0.90	-	-	-	-
	120	DL 59.5 (11.2)	DL 61.8 (8.4)	-	DL 0.88	-	-	-	-
	180	DL 50.5 (11.5)	DL 53.6 (9.1)	-	DL 0.89	-	-	-	-
	60	NDL 61.1 (9.0)	NDL 63.5 (10.6)	-	NDL 0.95	-	-	-	-
	120	NDL 55.2 (9.5)	NDL 60.0 (11.2)	-	NDL 0.86	-	-	-	-
	180	NDL 45.5 (11.1)	NDL 49.9 (6.7)	-	NDL 0.81	-	-	-	-
Iga et al. (2006)[5]	1,08 rad/s	DL 87 (24)	DL 92 (28)	-	-	-	-	-	-
	2,16 rad/s	DL 79 (20)	DL 85 (25)	-	-	-	-	-	-
	4,32 rad/s	DL 71 (17)	DL 73 (19)	-	-	-	-	-	-
	1,08 rad/s	NDL 84 (22)	NDL 89 (25)	-	-	-	-	-	-
	2,16 rad/s	NDL 75 (19)	NDL 77 (20)	-	-	-	-	-	-
	4,32 rad/s	NDL 68 (19)	NDL 67 (17)	-		-	-	-	-
Ayalon et al. (2000)[27]	90	14.69 (2.13)	16.41 (3.08)	-	ICC 0.95–0.98	-	-	-	-
Van den Berg-Emons et al. (1996)[21]	30	23.7 (9.1)	21.9 (10.0)	-	-	-	-	-	-
	60	21.2 (10.1)	20.2 (8.8)	-	-	-	-	-	-
	120	18.3 (8.2)	19.0 (7.8)	-	-	-	-	-	-

ICC = intraclass correlation coefficient (95% Confidence Interval); SEM = standard error of measurement; SRD = smallest real difference; DL = dominant leg; NDL = non dominant leg.

Only three articles [2, 4, 12] described the SEM. The values were represented in Newton/meters and as a percentage (Table 5).

**Table 5:** Eccentric extension and flexion of the knee in isokinetic, intra-class correlation coefficient, standard error of measurement and smallest real difference of strength measurements.

Knee action evaluated	Speed (°/s)	Mean /(SD) 1° test	Mean /(SD) 2° test	ICC (95%CI)	SEM (Nm)	SEM (%)	SRD (Nm)	SRD (%)
**ECCENTRIC**								
**EXTENSION**								
Fagher et al. (2016)[4]	60	64.3 (23.0)	63.3 (17.8)	0.70 (0.40–0.86)	11.5	18	31.8	49.7
Kellis et al. (1999)[11]	60	DL 130.5 (15.6)	DL 135.7 (22.7)	0.92	-	-	-	-
	120	DL 129.5 (23.8)	DL 125.5 (23.8)	0.88				
	180	DL 115.5 (16.1)	DL 121.9 (20.7)	0.80				
	60	NDL 127.3 (17.0)	NDL 136.1 (26.5)	0.82				
	120	NDL 131.1 (24.1)	NDL118.2 (16.9)	0.76				
	180	NDL 112.8 (14.7)	NDL 121.3 (21.2)	0.81				
Iga et al. (2006)[5]	2.16 rad /s	DL 182 (53) NDL 180 (47)	DL 194 (51) NDL 189 (51)	-	-	-	-	-
**FLEXION**								
Fagher et al. (2016)[4]	60	49.2 (19.6)	42.2 (12.0)	0.60 (0.22–0.81)	9.8	21.5	27.3	59.6
Kellis et al. (1999)[11]	60	DL 82.8 (10.5)	DL 80.6 (12.6)	0.85	-	-	-	-
	120	DL 82.4 (12.8)	DL 78.4 (11.0)	0.71	-	-	-	-
	180	DL 78.8 (11.2)	DL 81.0 (12.5)	0.76	-	-	-	-
	60	NDL 75.8 (10.4)	NDL 80.9 (16.7)	0.79	-	-	-	-
	120	NDL 80.9 (16.7)	NDL 75.9 (11.2)	0.79	-	-	-	-
	180	NDL 76.0 (8.9)	NDL 79.3 (14.6)	0.86	-	-	-	-
Iga et al. (2006)[5]	2.16 rad /s	DL 105 (28) NDL 98 (24)	DL 110 (29) NDL 102 (28)	-	-	-	-	-

ICC = intraclass correlation coefficient (95% Confidence Interval); SEM = standard error of measurement; SRD = smallest real difference; DL = dominant leg; NDL = non dominant leg.

The SRD was described in only two articles [4, 12]. These values were also represented in Newton/meters and as a percentage. All values were below 60% for flexion in the eccentric mode (Table 4).

There were many differences in the values of mean and standard deviation of peak torque (Tables 4 and 5). For example, in Fagher et al. [4], in the concentric mode, the values of the second measure were better than the first one for knee extension and for knee flexion. However, in the eccentric mode, the values of the second measure were worse than the first one for both knee extension and knee flexion.

Moreover, several studies found decreases in knee flexion in the concentric mode from the first to the second measurement [3, 5, 20, 21].

In the three articles using the eccentric mode [4, 5, 11], there were also differences in the values observed when comparing the test-retest.

### Meta-analysis

We performed a meta-analysis of the five studies for the reliability of knee movement in the concentric mode for both the extension and flexion of the joint of the subjects’ dominant leg at a speed of 60°/s.

In this aspect, the aforementioned reliability was observed in more detail. For the knee extension, the correlation coefficient was 0.89 for fixed effects and 0.90 for random effects (Table 6).

**Table 6:** Summary meta-analysis of reliability with confidence interval for extension and flexion of knee in children in concentric mode at 60°/s.

Extension						Weight (%)	
Study	Sample size	Correlation coefficient	95% CI	z	P	Fixed	Random
Fagher et al. (2016)[4]	22	0.810	0.590 to 0.918			23.46	21.64
Johnsen et al. (2015)[12]	28	0.870	0.736 to 0.938			30.86	23.18
Santos et al. (2013)[2]	21	0.810	0.582 to 0.920			22.22	21.31
Moreau et al. (2008)[10]	12	0.950	0.827 to 0.986			11.11	16.54
Kellis et al. (1999)[11]	13	0.980	0.933 to 0.994			12.35	17.32
Total (fixed effects)	96	0.888	0.832 to 0.926	12.721	<0.001	100.00	100.00
Total (random effects)	96	0.904	0.799 to 0.955	7.371	<0.001	100.00	100.00
**Flexion**							
Fagher et al. (2016)[4]	22	0.620	0.269 to 0.826			23.46	21.94
Johnsen et al. (2015)[12]	28	0.810	0.626 to 0.909			30.86	23.91
Santos et al. (2013)[2]	21	0.790	0.544 to 0.911			22.22	21.53
Moreau et al. (2008)[10]	12	0.960	0.860 to 0.989			11.11	15.88
Kellis et al. (1999)[11]	13	0.900	0.692 to 0.970			12.35	16.76
Total (fixed effects)	96	0.819	0.733 to 0.879	10.386	<0.001	100.00	100.00
Total (random effects)	96	0.838	0.694 to 0.918	6.626	<0.001	100.00	100.00

95% CI = 95% confidence interval.

For knee flexion, this correlation coefficient reached values of 0.82 for fixed effects and 0.84 for random effects.

**Fig 2:**
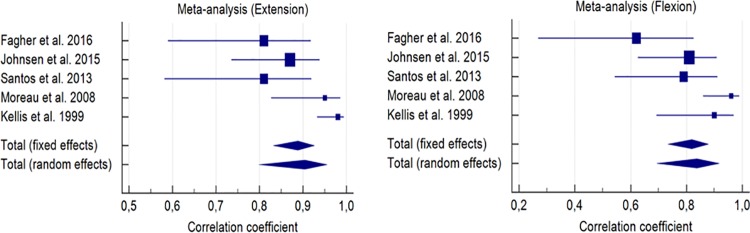
Reliability forest plot between evaluators (Bayes estimation) for each study, (random-effects model), with a 95% confidence interval for each reliability coefficient. Extension of knee (first figure) and knee flexion (second figure) in the concentric mode.

Fig 2 shows the values mentioned in the above table in a more schematic way. This figure allows quickly comparing each of the correlation coefficients, both for fixed effects and for random effects, between the concentric extension and flexion of the knee.

Regarding the heterogeneity of each action of the knee, we emphasize:

- For knee extension, a value of Q = 12.59; I^2^ = 68.22; Significance (*p*) = 0.0135; 95% of the IC for I^2^ = 18.05–87.68.
- For knee flexion, a value of Q = 10.29; I^2^ = 61.14%; Significance (*p*) = 0.0358; 95% of the IC for I^2^ = 0.00–85.41.

### Publication bias

**Fig 3:**
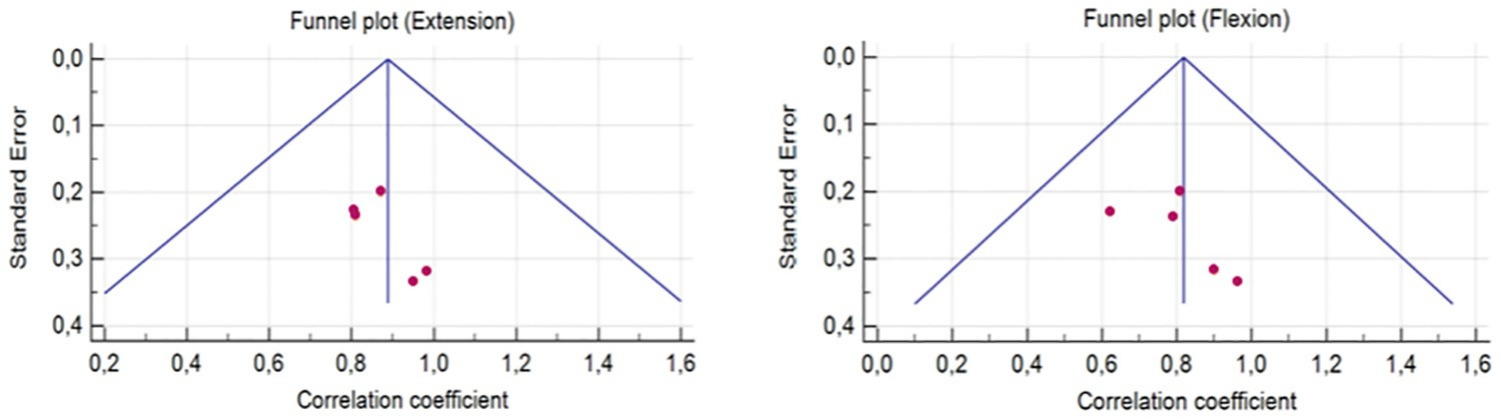
Funnel plot of extension and flexion.

Fig 3 shows funnel plots corresponding to knee extension and flexion in the studies.

The Egger test was used to evaluate the asymmetry of the funnel plot. The results of the Egger test for both knee extension and flexion were not significant (p = 0.109 and p = 0.136), suggesting that there is no publication bias.

## Discussion

The knee is one of the most studied joints in isokinetic dynamometry research, probably because it is easy to evaluate using a dynamometer [1]. Knee extensor and flexor strength are recognized as important for daily tasks, e.g., moving, standing or sitting, weight lifting, and climbing stairs [1]. In addition, many studies of knee extensors indicate that these could be representative of the total strength of the lower extremities. It is not only important to analyze the absolute values of flexors or extensors, but also the balance of both at the same time if the contralateral deficit is a compensated deficit; i.e., there is an adequate agonist/antagonist relationship [22]. Isokinetic dynamometry offers clear advantages over simple measurements to assess muscle strength, such as the continuous and accurate measurement of force production across a range of velocities of movement [23].

As we have indicated in the results, there were different heterogeneities for the test-retest data in each of the articles: they showed high and moderate values. Most authors thought that the results for the second tests would be better due to a learning effect [8, 24]. Some studies indicated a need for additional practice and familiarization with test procedures in this age group [25]. Other studies emphasized that the isokinetic method has the advantage of making it possible to detect large differences in the same muscles when analyzing at peak torque [26].

We analyzed the studies that reported reliability for the assessment of muscle strength in healthy children with CP. Our review suggests that the studies had good-to-excellent levels of reliability of muscle strength assessment in dominant and non-dominant knees using isokinetic dynamometry (0.7–1 correlation), except for Pierce et at. [20], which had low and moderate intra-class correlations at the velocities 15°/s and 90°/s, and Fagher et al. [4], where most results were moderate. Our results also support the contention that the muscle strengths of healthy children and children with CP are quantifiable and measurement is reliable, even in the presence of spasticity.

Otherwise, there were four studies whose participants were children with CP [10, 20, 21, 27] and one study whose participants were children without CP [11] where the values for the second test decreased compared to the first test in the concentric mode, but only for knee extension. The ICCs of these articles, except for Pierce et al. [20], were high for knee extension (Table 4). These results demonstrate that voluntary muscle fatigue of the knee flexors and extensors can be reliably and feasibly assessed in children. It is believed that the number of repetitions and the type of contraction may influence the assessment of peak voluntary torque in subjects with CP. A possible explanation for this could be that the coordination of the agonist and antagonist muscles is more impaired at higher velocities than at lower velocities in children with CP [21]. It is suggested that there is higher heterogeneity for children with CP than for their healthy peers. The tests may be less suitable for children with CP than for healthy children because of attention deficits, which are known to occur with CP [28]. Future research should establish normative values regarding spasticity of the knee flexion and knee extension in children with CP to allow for the clearest interpretations of clinically meaningful change in these subjects.

This review emphasizes the need to find a consensus around the minimum change necessary to indicate a clinically important change for an individual or a particular population. Analyzing the only two articles that included SRD [4, 12] led us to suggest that: 1) for the knee extension in the concentric mode, the minimum change is around 15–15.5 Nm in absolute value and 31% in relative value with a speed of 60°/s and 34.5% with a speed of 180°/s; in the eccentric mode, the SRD should be 49.8%. 2) For knee flexion in the concentric mode, the minimum change is around 9.8–13.0 Nm in absolute value and 36.5–38.5% in relative value; in the eccentric mode, the SRD should be 59.6%.

Most of the studies had a time interval of one week between tests. One study had a three-days interval [3] and another two studies had time intervals lower than two hours [20, 21]. It is reasonable to establish a one-week interval because intervals of approximately 1 week between tests could maximize the effects of learning while still managing any effects of muscle fatigue [29].

A meta-analysis evaluates the replicability and generalizability of results, which are the hallmarks of good science [30]. This review presents a meta-analysis for the reliability of each of the included studies. The results of our analyses are shown in Tables 3 and 4. We hope to provide more accurate and definitive values that will guide future research on the population treated here. Table 6 shows that the reliability varies. However, reliability is high for all the studies analyzed: for knee extension, reliability reaches a high—good level (0.89) for fixed effects and an excellent—very high level (0.90) for random effects; for knee flexion, reliability reaches a high—good level (0.82–0.84) for both fixed and random effects, which are lower values than for extension. The study of Kellis et al. [11] is the most reliable for extension, with an intra-class correlation coefficient of 0.98 and a range of 0.933–0.994 for 95% of the CI. For flexion movement, Moreau et al.’s study [10] meets the high expectations seen in Tables 4 and 5 for articles dealing with subjects with CP: this article has a correlation coefficient of 0.96 and a range of 0.860 to 0.989 for 95% of the CI. According to the meta-analysis, the relative reliability is high.

The methodological quality of this review, shown in Tables 1 and 2, means it is a very useful tool for comparing the studies and allows drawing conclusions about the reliability of the studies. First, the percentages of items met (Tables 1 and 2) indicate the limitations of the articles depending on the requested variables. Only one article reaches 78% quality [12], in CAT scale, and three are below 50%. The final mean of the percentages is approximately 53%, meaning that only half of the variables are fulfilled. In QAREL scale only one article reaches 100% quality and eight articles are above 55%.

The meta-analysis shows that one study has a 78% (CAT scale) and 100% (QAREL scale) score for methodological quality [12], another had 67% and 70% [2], and three studies had 56% and 60% [4, 10, 11]. The meta-analysis (5 studies) has a small sample (n = 96), but these results seem very interesting, since the reported reliability coefficients are high and excellent. High reliability coefficients are usually reported by studies with small sample sizes, which are associated with large standard errors and higher than normal CIs [30].

### Limitations

The main limitation is the heterogeneity (isokinetic devices, populations, protocols…) of the identified studies. Because of the relatively small numbers of eligible studies, and the variability in the statistics used between studies, there were insufficient numbers to allow a meta-analysis for all velocities and the eccentric mode. For the same reason, a sub-analysis of the possible subgroups could not be performed. Of the five articles selected, four treated healthy children and only one study evaluated children with cerebral palsy. As for another possible subgroup, two articles evaluated soccer-playing children and three evaluated children who were not soccer players. As stated above, one evaluated children with cerebral palsy.

Publication bias exists in any literature review and should be considered when interpreting the results. Measures were taken to minimize bias as much as possible, including conducting a comprehensive search of multiple databases with gray literature databases. Only articles published in English and in peer-reviewed journals were included, which may have reduced the number of results. In addition, this meta-analysis was not registered online.

## Conclusions

The present review and meta-analysis explores existing data about different results in the test-retest reliability of isokinetic knee strength measurements in healthy and with CP children. This study provides a reliable analysis of isokinetic knee force measurements in the concentric mode. These findings suggest that isokinetic dynamometry can be used not only for measuring muscle strength for training, but also for muscle strength reeducation programs in children with or without CP.

More studies are needed in children with different pathologies to confirm our results. In addition, future research should evaluate knee force in the eccentric mode and show the reliability of test-retest measurements.

## Supporting information

S1 File PRISMA checklist. (DOC)
